# Glucagon-like peptide-1 (GLP-1) receptor agonists and cardiovascular events in patients with type 2 diabetes mellitus: a meta-analysis of double-blind, randomized, placebo-controlled clinical trials

**DOI:** 10.1186/s12902-022-01036-0

**Published:** 2022-05-12

**Authors:** Jing Qin, Li Song

**Affiliations:** 1 Department of Emergency, The Second hospital of Lanzhou University, Lanzhou, China; 2 Department of Internal Medicine 1 and Clinical Chemistry, University Hospital of Heidelberg, Heidelberg, Germany

**Keywords:** Glucagon-like peptide-1, Type 2 diabetes mellitus, Cardiovascular events

## Abstract

**Background:**

The cardiovascular effects of glucagon-like peptide-1 (GLP-1) receptor agonists are still controversial in the treatment of type 2 diabetes mellitus (T2DM) patients. The purpose of this study was to evaluate the risk of cardiovascular events of GLP-1 (albiglutide, exenatide, liraglutide, semaglutide, lixisenatide and dulaglutide) receptor agonists in T2DM patients.

**Methods:**

PubMed and Embase were searched to find relevant randomized controlled trials (RCTs) from inception to June 2019 that evaluated the effect of GLP-1 receptor agonists on cardiovascular events in patients with T2DM. The T2DM patients of all the eligible trials received either GLP-1 therapy or placebo, and the cardiovascular outcomes included death from cardiovascular causes, fatal or non-fatal myocardial infarction and fatal or non-fatal stroke.

**Results:**

We included 6 multinational double-blind randomized placebo-control trials that included a total of 52821 T2DM patients. The results indicated that GLP-1 receptor agonists reduced the risk of death from cardiovascular causes (RR: 0.90; 95% CI: 0.83–0.97; *P* = 0.004) and fatal or non-fatal stroke (RR: 0.85; 95% CI: 0.77–0.94; *P* = 0.001) compared with the placebo controls. But GLP-1 receptor agonists did not significantly alter the fatal or non-fatal myocardial infarction compared with the placebo (RR: 0.91; 95% CI: 0.82 – 1.01; *P* = 0.06).

**Conclusion:**

We concluded that GLP-1 receptor agonist therapy could reduce the risk of death from cardiovascular causes and fatal or non-fatal stroke compared with the placebo in the treatment of T2DM patients in trials with cardiovascular outcomes.

## Background

Type 2 diabetes mellitus patients have a very high risk of cardiovascular events, including death from cardiovascular causes, fatal or non-fatal myocardial infarction and fatal or non-fatal stroke [1]. The rates of cardiovascular death are 2- to 4-fold higher for patients with diabetes compared with the rates for those without diabetes [2]. GLP-1 receptor agonists, which as the glucose-lowering therapeutic agents in the treatment of type 2 diabetes mellitus, have been shown to affect the incidence of cardiovascular outcomes in patients with type 2 diabetes mellitus, although the results regarding GLP-1 receptor agonists remain inconsistent [3, 4]. It is well known that GLP-1, as a peptide hormone, stimulates insulin secretion and inhibits glucagon secretion in a glucose-dependent manner [5]. An increasing number of studies have shown that glucagon-like peptide-1 (GLP-1) may improve endothelial functioning and may have direct effects in protecting the vascular system [6]. There are several GLP-1 receptor agonists that are used as therapeutic agents for treating type 2 diabetes mellitus patients in clinical fields. Recently, the GLP-1 receptor was believed to have an effect on individual cardiovascular outcomes in the treatment of diabetes, but not all GLP-1 receptor agonists showed the effect of reducing cardiovascular outcomes because of the varied effectiveness of the different GLP-1 drugs. Just like some multinational randomized controlled trials elaborated that the use of GLP-1 receptor agonists to reduce the rate of cardiovascular events in T2DM patients [7–10]. While other clinical studies concluded that the GLP-1 receptor agonists did not significantly alter the major cardiovascular outcomes in patients with type 2 diabetes [3, 4]. Moreover several meta-analysis had concentrated on the cardiovascular effects and the safety in GLP-1-treated T2DM patients [11, 12], but the conclusion were inconsistent.

Therefore, we performed a meta-analysis of double-blind randomized placebo-controlled clinical trials to investigate the cardiovascular complications of GLP-1 receptor agonists in T2DM patients. The cardiovascular outcomes included death from cardiovascular causes, fatal or non-fatal myocardial infarction and fatal or non-fatal stroke.

## Methods

### Data sources and search strategy

We comprehensively searched PubMed and Embase to find relevant randomized controlled trials (RCTs) from inception to June 2019 that evaluated the effect of GLP-1 receptor agonists on cardiovascular events in patients with T2DM. This meta-analysis was conducted and reported in accordance with the 2009 Preferred Reporting Items for Systematic Reviews and Meta-Analysis Statements [13]. We adhere to the PRISMA guidelines in this meta-analysis [13]. The language was confined to English. The search terms were as follows: “incretin” OR “GLP-1” OR “glucagon-like peptide-1 analogue” OR “Liraglutide” OR “exenatide” OR “liraglutide” OR “lixisenatide” OR “albiglutide” OR “dulaglutide” OR “semaglutide” OR “taspoglutide” AND “type 2 diabetes mellitus” OR “T2DM” AND “randomized controlled trials” OR “RCT”. We also comprehensively screened the references of reviews and articles in order to find more eligible articles.

### Data selection criteria

The literature search was screened independently by two authors; if there were some inconsistencies, we discussed within the group until a consensus was reached. The research titles and abstracts were initially screened, and then we screened the study design, interventions, control, and outcomes in detail to determine the included trials.

The criteria for including eligible studies were as follows: (1) the studies were double-blind, randomized placebo-controlled trials; (2) the RCTs were evaluating GLP-1 versus placebo in T2DM patients; (3) there was a comparison of cardiovascular risk between GLP-1 receptor agonists and placebo in T2DM patients with or without cardiovascular diseases; and (4) a risk ratio (RR) with corresponding 95% confidence intervals (CIs) or data was reported.

Regarding the exclusion criteria, we excluded studies with the following criteria: (1) the case and control patients; (2) studies with irrelevant data and the small sample size trials which contain less than 3000 T2DM patients; and (3) duplicate publications, animal experimental studies, reviews, conference abstracts, or meta-analyses.

### Data extraction and quality assessment

The following data were extracted from the included RCTs by two authors independently: first author’s name; publication year, country, sample size, study design, intervention, glycated haemoglobin, duration of diabetes and the follow-up periods. The quality of the included studies was assessed by the Cochrane Collaboration tool for the risk of bias [14]. Each studies was identified as “low,” “high” or “unclear” risk of bias based on the following items: the statement of randomization, blinding, details of withdrawals and dropouts, generation of random numbers, and concealment of allocation. The quality of each results was assessed according to the Grading of Recommendations, Assessment, Development, and Evaluation (GRADE) system which classifies the quality of evidence as high, moderate, low or very low [15].

### Data synthesis and statistical analysis

This meta-analysis was performed using Review Manager 5.3 software (RevMan), The Cochrane Collaboration, Copenhagen. For the cardiovascular events in our study, we calculated the risk ratio (RR) with 95% confidence intervals (CIs) to standardize the differences between the GLP-1 receptor agonist and placebo. The forest plots were conducted using a fixed-effect model if there was no obvious heterogeneity or using a random-effect model when heterogeneity of the included studies was obvious [16, 17]. Additionally, the chi-squared (χ^2^) test and the I^2^ test were used to assess the heterogeneity between studies. When *P* ≤ 0.10 and I^2^ > 50%, the heterogeneity between those included studies was defined as obvious heterogeneity [18]. Moreover, if the I^2^ test value was 25–50%, it was defined as mild heterogeneity, 50–75% as moderate heterogeneity, and 75% as severe heterogeneity. To measure publication bias, we performed a funnel plot and Egger’s tests. A funnel plot was used to qualitatively measure the publication bias [19, 20], and *P* ≤ 0.05 was considered significant publication bias in this meta-analysis.

## Results

**Fig. 1:**
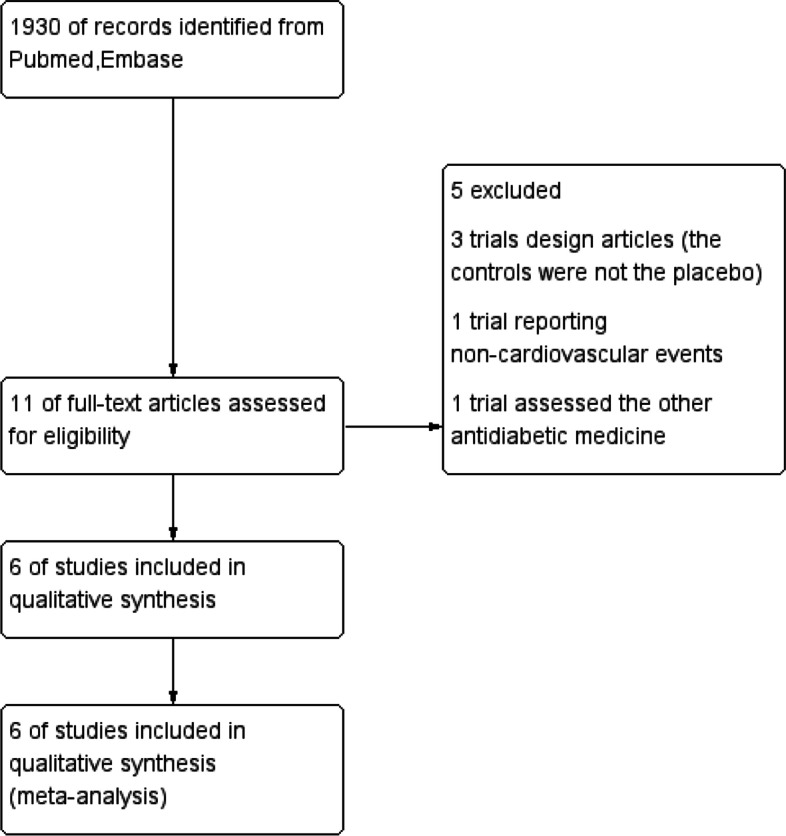
The flow diagram of the included studies

**Fig. 2:**
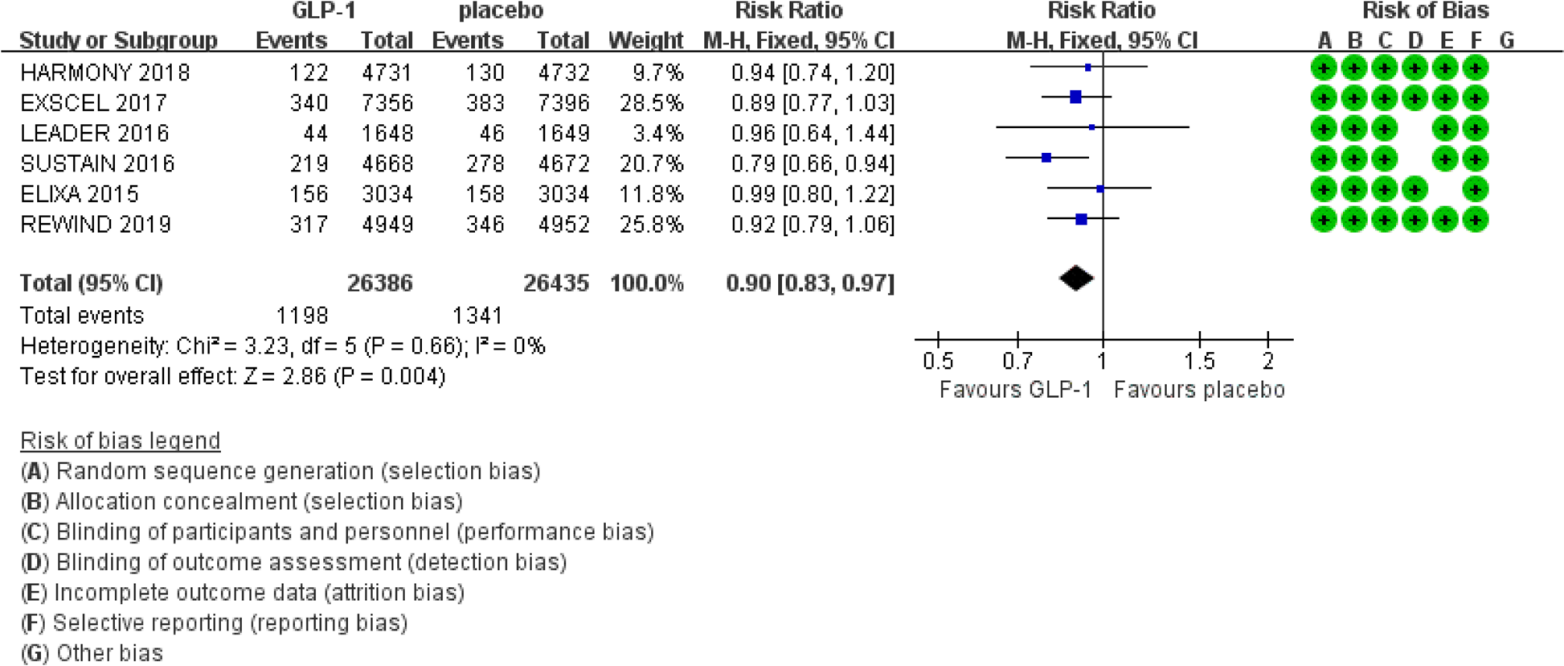
Effect of GLP-1 versus placebo on death from cardiovascular causes

**Fig. 3:**
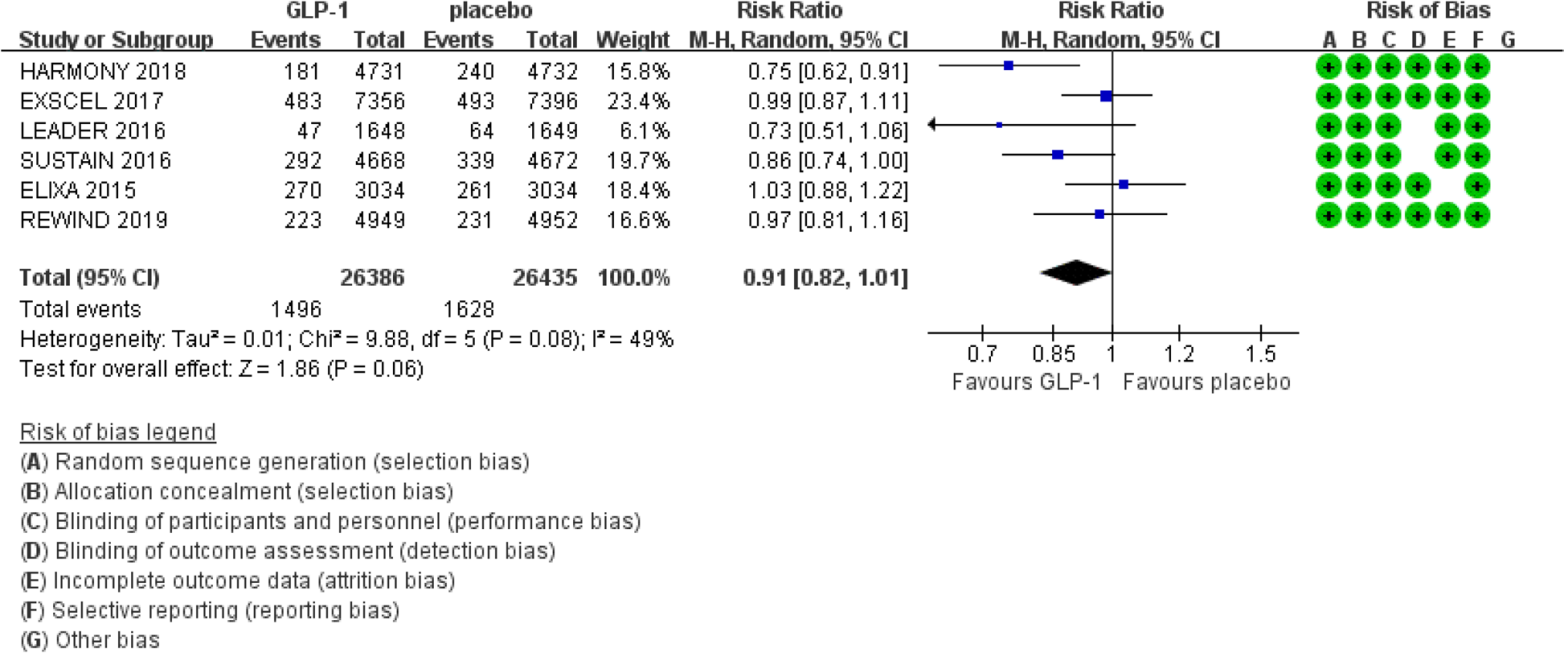
Effect of GLP-1 versus placebo on fatal or non-fatal myocardial infarction

**Fig. 4:**
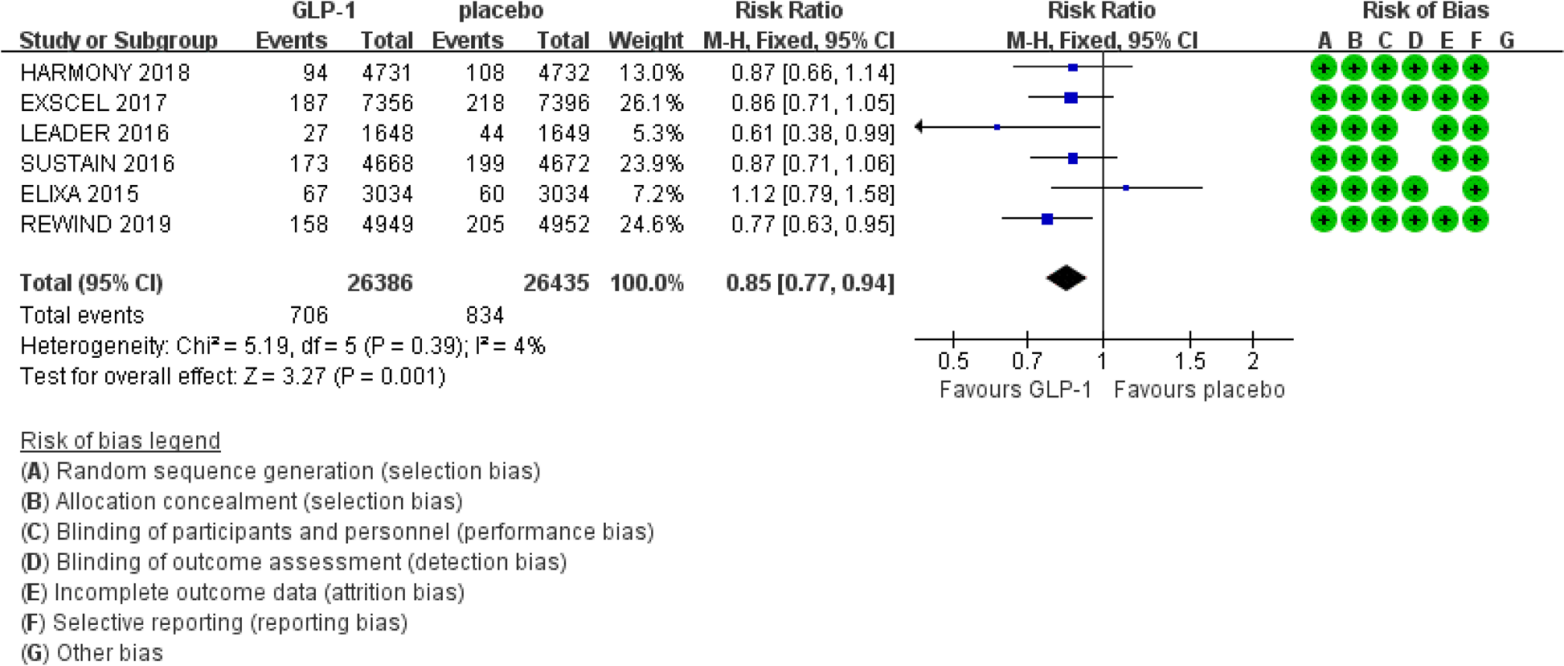
Effect of GLP-1 versus placebo on fatal or non-fatal stroke

We obtained 1930 articles after searching PubMed and Embase from 2011 to June 2019. Then, we screened the titles and abstracts and removed the duplicate articles, reviews and conference abstracts, and 11 articles remained for evaluating the details of the full text to determine whether they met the inclusion criteria. Finally, 6 trials were included in this meta-analysis (Fig. 1) [3, 4, 7–10]. The Cochrane Collaboration tool was applied to evaluate the quality of the included trials. The results regarding the individual quality of the included trials are shown in Fig. 2,3,4.

The selected studies were published between 2015 and 2019. The GLP-1 receptor agonist arms included 26386 patients, and the placebo control arms included 26435 patients. The main characteristics of the included trials are presented in Table 1. The control treatment in the included trials was placebo according to the experimental trial treatment. The cardiovascular outcomes included death from cardiovascular causes, fatal or non-fatal myocardial infarction and fatal or non-fatal stroke.

**Table 1:** The characteristics of included trials

Study	Country of publication	Country of patients	Experimental Sample size	Control Sample Size	Age	Duration of diabetes	Intensive Therapy	Control therapy	Follow-up
Adrian F Hernandez 2018 [10]	USA	Western Europe Eastern and central Europe North America Latin America Asia Pacific	4731	4732	64.2/ 64.2	4.1/ 4.2	Albiglutide (30-50mg) once a week	Placebo once a week	1.5 years
Rury R. Holman 2017 [4]	United Kingdom	Europe, Latin America Europen subcategories, Asia-pacific, Eastern Europe, Western Europe, North America	7356	7396	<65y 8813 ≥65y 5939	12.0 (7.0-18.0)	exenatide at a dose of 2 mg once weekly	Placebo once a week	2.3 years
Steven P2016 [7]	USA	North AmericaEurope Asia Rest of the world	4668	4672	<60y 2321 ≥60y 7019	12.8 years	liraglutide 1.8 mg (or the maximum tolerated dose) once daily	placebo once daily	3.8 years
Steven P. Marso2016 [8]	United Kingdom	230 sites in 20 countries.	1648	1649	64.6± 7.4	13.9± 8.1	semaglutide (0.5mg or 1.0mg) once a week	Placebo once a week	109 weeks
Preffer MA 2015 [3]	Thailand	49 countrirs	3034	3034	60.3	9.3	lixisenatide	placebo	2.1 years
Gerstein HC 2019 [9]	UK	371 sites 24 countries	4949	4952	66.2	10.5/ 9.5	dulaglutide 1.5mg	placebo	5.4 years

There was no obvious heterogeneity in the six included studies (I^2^ = 0%, Cochran Q test *P* = 0.66) (Fig. 2) regarding the risk of death from cardiovascular causes. Therefore, we used the fixed effect model in the RevMan software. GLP-1 receptor agonists reduced the risk of death from cardiovascular causes compared with the placebo (RR: 0.90; 95% CI: 0.83 – 0.97; *P* = 0.004) according to the results of the meta-analysis.

There was mild heterogeneity (I^2^ = 49%, Cochran Q test *P* = 0.08) (Fig. 3) regarding the risk of fatal or non-fatal myocardial infarction in the included studies. Therefore, we used the random effect model in the RevMan software. No significant effect of GLP-1 receptor agonists identified on the risk of fatal or non-fatal myocardial infarction compared with the placebo controls (RR: 0.91; 95% CI: 0.82 – 1.01; *P* = 0.06).

There was also no evidence of heterogeneity observed across the included trials regarding fatal or non-fatal stroke (I^2^ = 4%, Cochran Q test *P* = 0.39) (see Fig. 4). The fixed effect model was applied in the RevMan software. The results of the meta-analysis indicated that GLP-1 receptor agonists reduced the risk of fatal or non-fatal stroke compared with the placebo (RR: 0.85; 95% CI: 0.77 – 0.94; *P* = 0.001).

Because only six trials were included, which is less than ten, we have no evidence of publication bias in this meta-analysis by the funnel plot. No individual study had a significant effect on the pooled effect size according to the results of the sensitivity analysis at all end points.

## Discussion

The objective of this meta-analysis was to explore the effect of GLP-1 receptor agonists on cardiovascular outcomes in type 2 diabetes mellitus patients. The results of this meta-analysis suggest that GLP-1 therapy has a significant impact on the incidence of death from cardiovascular causes and fatal or non-fatal stroke in T2DM patients. There was no heterogeneity in these six included studies in their assessment of the effect of GLP-1 receptor agonists on the risk of death from cardiovascular causes and fatal or non-fatal stroke; however, there was mild heterogeneity regarding the risk of fatal or non-fatal myocardial infarction in the included studies, and the reasons may be the specific medicine molecule and GLP-1 receptor agonist dose tested, differences in the randomized patients (such as medical history and baseline characteristics), duration of follow-up years and adherence to treatment. As our findings are based on good-quality studies that were all multinational double-blind randomized placebo-control trials and our meta-analysis was based on a mean follow-up of 3.45 years (minimum 1.5 year – maximum 5.4 years), the confounding and attrition bias are controlled, so the risk of unreliable results is diminished.

Similar to our results, some studies [21, 22] also indicated that GLP-1 receptor agonists had a positive effect on the heart and enhanced cardiac function. While some previous studies [23] found a lower incidence of cardiovascular disease (CVD) events when GLP-1 receptor agonists were compared with placebo. Furthermore, a comparison meta-analysis [24–26] indicated that there was no significant reduction in CVD events by GLP-1 receptor agonists. Similarly, Inzucchi et al [27] believed that the evidence of GLP-1 receptor agonist cardiovascular protection was still limited, and the cardiovascular system benefits related to GLP-1 receptor agonists may be independent of its glucose, lipid, or energy metabolism effects. In order to better define the cardiovascular effects of GLP-1 receptor agonist in type 2 diabetes, we perform this meta-analysis. The trials included in the meta-analysis are the EXSCEL study (exenatide), LEADER study (liraglutide), SUSTAIN 6 study (semaglutide) and HARMONY outcomes study (albiglutide), ELIXA study (lixisenatide) and REWIND study (dulaglutide). Not all GLP-1 receptor agonist may have the same effect on cardiovascular events, since the classification of Glucagon-like peptide-1 (GLP-1) receptor agonists included the short acting and long acting. But there were no hand-to-hand cardiovascular outcomes studies focused on GLP-1 receptor agonist class, limiting the investigation of cardiovascular events differences between GLP-1 receptor agonists of different mechanical structure or drug potency.

GLP-1 receptor agonists have had cardiovascular protection effects in cardiovascular trials, the cardiovascular protection mechanisms of GLP-1 RAs contain direct and indirect effects which have been well discussed in preclinical and clinical studies [28]. The cardiovascular indirect effects of GLP-1 receptor agonists might be mediated via improve the common cardiovascular metabolic risk factors such as HbA1c, systolic blood pressure, and body weight, anti-inflammatory pathways, ischaemic conditioning and endothelial function [12, 28–30]. In addition, the direct effects of GLP-1 RAs to the cardiovascular system included enhancing the endothelial function, cardiac output, and myocardial glucose uptake, since the abundant GLP-1 receptor expressed in cardiac and vascular tissues [28, 30].

This meta-analysis has several strengths: (1) Only RCTs were included, so this meta-analysis eliminated the potential control group biases; (2) The large sample size of the 6 included trials allowed us to quantitatively evaluate the GLP-1 receptor agonist effects in T2DM patients; (3) A wide range of patient characteristics was represented, which ensured a comprehensive assessment of the effect of GLP-1 receptor agonists in the treatment of patients with T2DM.

This study has some limitations. First, the publication bias is an inevitable problem in any meta-analysis. Second, T2DM patients who received various GLP-1 receptor agonist drugs, such as albiglutide, exenatide, liraglutide, semaglutide, lixisenatide and dulaglutide might have biased the meta-analysis results. Third, a more detailed analysis was restricted because the meta-analysis used pooled data.

## Conclusion

The findings of this study indicated that GLP-1 receptor agonist therapy reduced the incidence of death from cardiovascular causes and fatal or non-fatal stroke in the treatment of T2DM patients. We need additional large RCTs in the future to evaluate the treatment effects of GLP-1 receptor agonists in T2DM.

## Data Availability

The datasets generated or analyzed during the current study available from the corresponding author on reasonable request.

## Abbreviations

GLP-1 RAs: Glucagon-like peptide 1 receptor agonists
T2DM: type 2 diabetes mellitus
RCTs: randomized controlled trials
GRADE: Grading of Recommendations, Assessment, Development, and Evaluation
CVD: cardiovascular diseases
RR: risk ratio
Cis: confidence intervals
HbA1c: glycosylated hemoglobin
